# Efficacy of Ultrasound-Guided Steroid Injections in the Management of Morton’s Neuroma: A Retrospective Cohort Study

**DOI:** 10.7759/cureus.96217

**Published:** 2025-11-06

**Authors:** Chi Hoi Lee, Balvinder Rana, Elliot Lee, Ahmed Zainy, Oliver M Scarborough, Madeline Websper, Anand Pillai

**Affiliations:** 1 Trauma and Orthopaedics, Manchester University NHS Foundation Trust, Manchester, GBR; 2 Trauma and Orthopaedics, Stockport NHS Foundation Trust, Manchester, GBR; 3 Trauma and Orthopaedics, Wythenshawe Hospital, Manchester University NHS Foundation Trust, Manchester, GBR; 4 Trauma and Orthopaedics, London North West University Healthcare NHS Trust, London, GBR

**Keywords:** corticosteroid injections, foot pathology, morton’s neuroma, pain, visual analogue scale

## Abstract

Background: Morton’s neuroma, a common painful neuropathy of the forefoot, is characterised by fibrotic thickening of plantar digital nerves. Patients often present with forefoot symptoms such as pain, burning, tingling or numbness, typically at the third web space. The exact cause of Morton’s neuroma is not fully understood. The most accepted theory suggests that Morton’s neuroma is caused by chronic repetitive microtrauma. Ultrasound (US)-guided corticosteroid injections can be used for symptom relief, but their pain-relieving efficacy is still unclear.

Methods: This retrospective cohort study evaluated the efficacy of US-guided corticosteroid injections in treating pain associated with Morton’s neuroma, and whether the neuroma size influences pain severity or response following corticosteroid injection. The data captured via SECTRA IDS7 (Sectra AB, Linköping, Sweden) were organised into the following categories: age, sex, smoking status, neuroma size and pre-injection and post-injection pain scores. Variables were analysed through statistical tests including analysis of variance (ANOVA), Spearman’s rank correlation coefficient and the Wilcoxon signed-rank test coefficient.

Results: Data from 20 eligible patients demonstrated that US-guided corticosteroid injections are 85% effective in controlling pain associated with Morton’s neuroma. The median duration of pain relief was 17 months, and the interquartile range (IQR) was 18 months (Q1=6, Q3=24). The mean visual analogue scale (VAS) scores decreased from 7.7 before injection to 2.2 after injection (P=0.006). The size of the neuroma at the time of the US scan was not related to pain severity or outcomes in terms of reduction in pain score (P=0.767). Female sex was associated with a greater reduction in the VAS pain score; however, this difference was not statistically significant (P=0.33). A P-value of less than 0.05 was used to determine significance. Six (30%) of the patients needed downstream surgery, which indicated the degree of recurrence or severity of pain.

Conclusions: US-guided corticosteroid injections are safe, effective and reasonable treatment choices for Morton’s neuroma, with variable durations of relief. No complications were observed in our study group following the intervention. Further research using a larger patient cohort is needed. This is to determine whether there are any associations between the efficacy of pain reduction in female patients and that in male patients. It will also assess whether neuroma size may be related to corticosteroid treatment response and highlight those who may benefit from earlier surgery.

## Introduction

Morton’s neuroma is a painful plantar digital neuropathy affecting the web spaces of the foot. Despite its name, it is not a true neuroma but rather a benign condition in which there is fibrotic thickening of the nerve. Patients often present with forefoot symptoms such as pain, burning, tingling or numbness, typically at the third web space [1-3]. The exact cause of Morton’s neuroma is not fully understood. The most accepted theory suggests that Morton’s neuroma is caused by chronic repetitive microtrauma. Other theories include nerve ischemia, intermetatarsal bursitis and nerve compression or entrapment [4,5].

Morton’s neuroma commonly affects those aged between 40 and 60 years, occurring in women more than in men. Data from the primary care setting in the United Kingdom reports the incidence as 88 per 100,000 women compared with 50 per 100,000 men. This makes Morton’s neuroma the second most common neuropathy after carpal tunnel syndrome [1,2].

The diagnosis is primarily clinical, with palpation of the affected area reproducing symptoms and compression of the metatarsals potentially eliciting an audible Mulder’s click, which supports the diagnosis [3,4]. An ultrasound (US) scan can detect Morton’s neuroma with 95% sensitivity, and magnetic resonance imaging (MRI) can further evaluate and rule out any uncertainty or equivocal US scan results [6].

Various treatment options exist for Morton’s neuroma, including modified footwear or orthotics, steroid injections, neuroma excision, neurolysis and nerve transposition [4-6]. Steroid injections around the affected nerve are commonly utilised, but their pain-relieving efficacy, duration of pain relief and relationship with neuroma size and injection outcome remain unclear [3]. Repeated injections can lead to reduced efficacy as well as side effects such as atrophy of the fat pad, skin damage or skin depigmentation [7,8]. This study investigated the effectiveness of US-guided corticosteroid injections in the treatment of Morton’s neuroma.

The aim of this study was to evaluate the pain-relieving efficacy of ultrasound-guided corticosteroid injections and explore associations with neuroma size and sex. This was assessed by analysing improvements in the visual analogue score (VAS) [9-11]. We also reported the number of injections given in each patient and how many patients ultimately underwent surgical excision after steroid injections.

## Materials and methods

Study design and data collection

**Figure 1:**
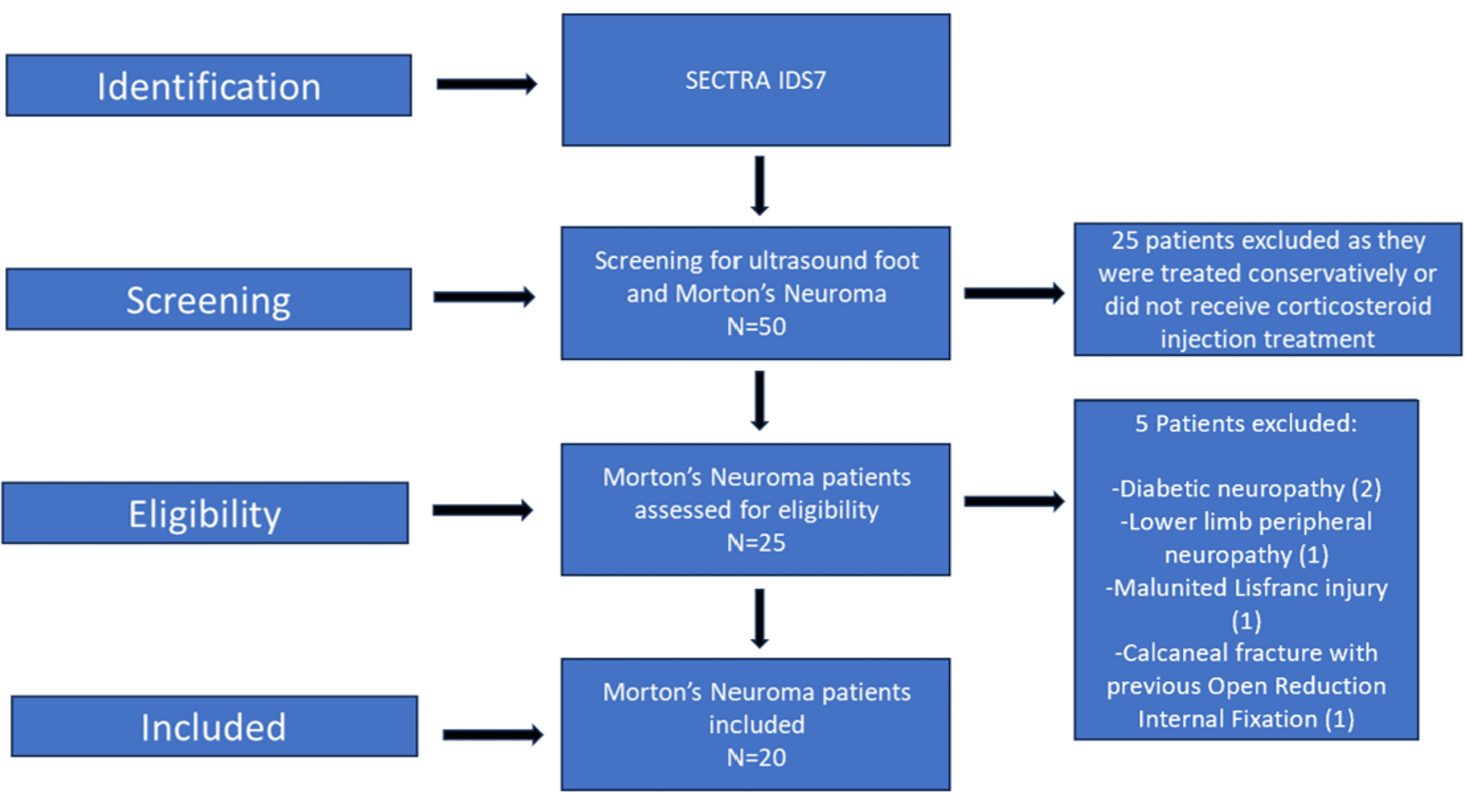
Flow diagram of patient selection

This was a retrospective observational cohort study conducted in the Department of Orthopaedics, Wythenshawe Hospital, Manchester University Foundation Trust. We searched the department’s radiology software SECTRA workstation IDS7 (Sectra AB, Linköping, Sweden) using the search criteria of “Ultrasound foot” and “Morton’s neuroma” to find patients who had a diagnosis of Morton’s neuroma on US reports and who later underwent US-guided steroid injections between January 2019 and January 2024. This study period was chosen to include a continuous five-year dataset to maximise case inclusion. Data were collected from the hospital’s electronic patient record system EPIC Hyperspace: HIVE, including age, sex, site and size of neuroma, number of injections, post-injection surgery, medical comorbidities, local pathology and smoking history. Patients with diabetes and peripheral neuropathy or patients who had concomitant foot pathologies were excluded. Figure 1 illustrates the process of patient selection. Corticosteroid injection treatment will be defined as follows: injection of 20 mg of triamcinolone acetonide (brand name: Kenalog) with 0.25% bupivacaine local anaesthetic in a 2 mL volume.

We interviewed all patients via telephone to document their pre-injection and post-injection pain scores via the VAS. The interview questionnaires were conducted with a minimum 24-month period following their most recent corticosteroid injection. They were asked to rate the maximum post-injection relief they obtained from the injection, the time after they experienced such relief and their current levels of pain.

The data were then analysed to assess the outcomes.

As per the NHS Health Research Authority guidance, this study did not require NHS Research Ethics Committee (REC) review for sites in England. This study has been prepared and reported in accordance with the Strengthening the Reporting of Observational Studies in Epidemiology (STROBE) guidelines for observational studies.

Statistical analysis

The data captured via SECTRA IDS7 were organised into the following categories: age, sex, smoking status, neuroma size and pre-injection and post-injection pain scores. Normality was assessed using the Shapiro-Wilk test, confirming non-normal distribution (P<0.05). Variables were then analysed through a series of statistical tests, including analysis of variance (ANOVA), Spearman’s rank correlation coefficient and the Wilcoxon signed-rank test coefficient. Results were considered statistically significant if the P-value was less than 0.05.

## Results

The initial screening with SECTRA IDS7 identified 25 patients who underwent US-guided steroid and local anaesthetic injection for Morton’s neuroma. Two of these patients had associated diabetic neuropathy; one patient had lower limb peripheral neuropathy of undetermined cause; one patient had a malunited Lisfranc injury and another patient had a previous calcaneal fracture open reduction internal fixation. Excluding these five patients, our study included a group of 20 patients who did not have any medical comorbidities or any other local foot pathology and who received a steroid injection for the neuroma.

Table 1 shows the master chart for all 20 patients included in the study, with the relevant data captured.

**Table 1:** Master chart of the study patients VAS: visual analogue scale

Patient number	Age	Size of neuroma (mm)	Pre-injection VAS	Post-injection VAS at maximum relief	Multiple corticosteroid injection	Total number of injections	Duration of gap between injections (months)	Surgical excision
1 (female)	56	3.9 (left), 3.5 (right)	8 (left and right)	4 (left and right)	Yes	2	19	Yes (listed for surgery)
2 (male)	35	2.6 (left)	9	0	No	1	-	No
3 (female)	60	3 (right)	7	0	No	1	-	No
4 (female)	65	8.2 (right)	8	5	Yes	2	19	No
5 (female)	28	8 (left)	10	0	Yes	2	4	Yes
6 (male)	72	4 (right)	4	4	Yes	2	5	No
7 (female)	76	4 (left)	10	0	No	1	-	No
8 (male)	53	4 (left)	6	6	Yes	2	6	Yes
9 (male)	37	5 (left)	6	0	Yes	2	30	No
10 (female)	54	3 (right)	7	3	No	1	-	No
11 (female)	76	5 (left)	8	0	Yes	3	36	No
12 (female)	63	5 (right)	10	0	Yes	2	6	No
13 (female)	30	5 (left)	2	0	No	1	-	No
14 (female)	56	4 (left)	8	0	No	1	-	Yes
15 (female)	51	3 (right)	6	0	No	1	-	No
16 (female)	72	4 (right)	8	5	No	1	-	No
17 (female)	68	4 (left)	9	9	No	1	-	Yes
18 (female)	63	3 (left)	10	2	Yes	2	15	No
19 (female)	69	3 (left)	8	2	No	1	-	Yes
20 (male)	75	4 (left)	10	4	Yes	2	24	No

Demographics

**Figure 2:**
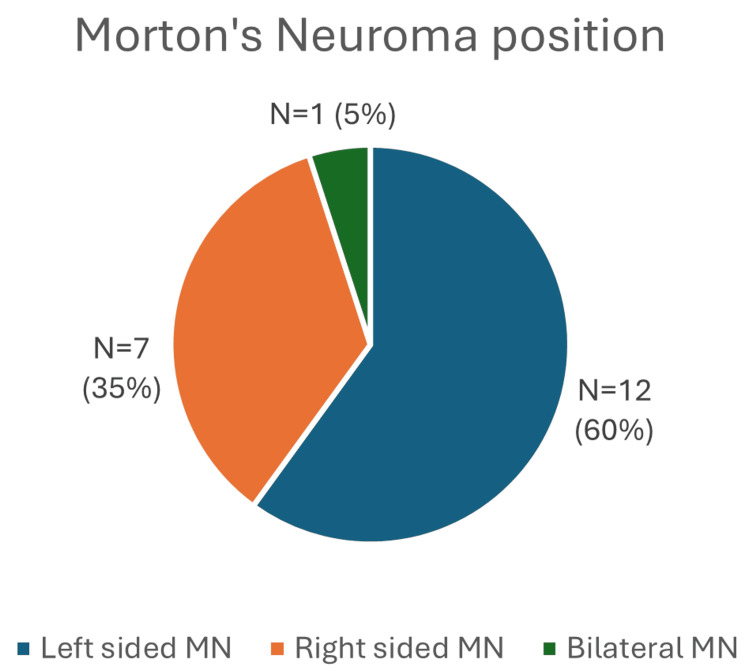
Chart showing Morton’s neuroma position MN: Morton’s neuroma

**Figure 3:**
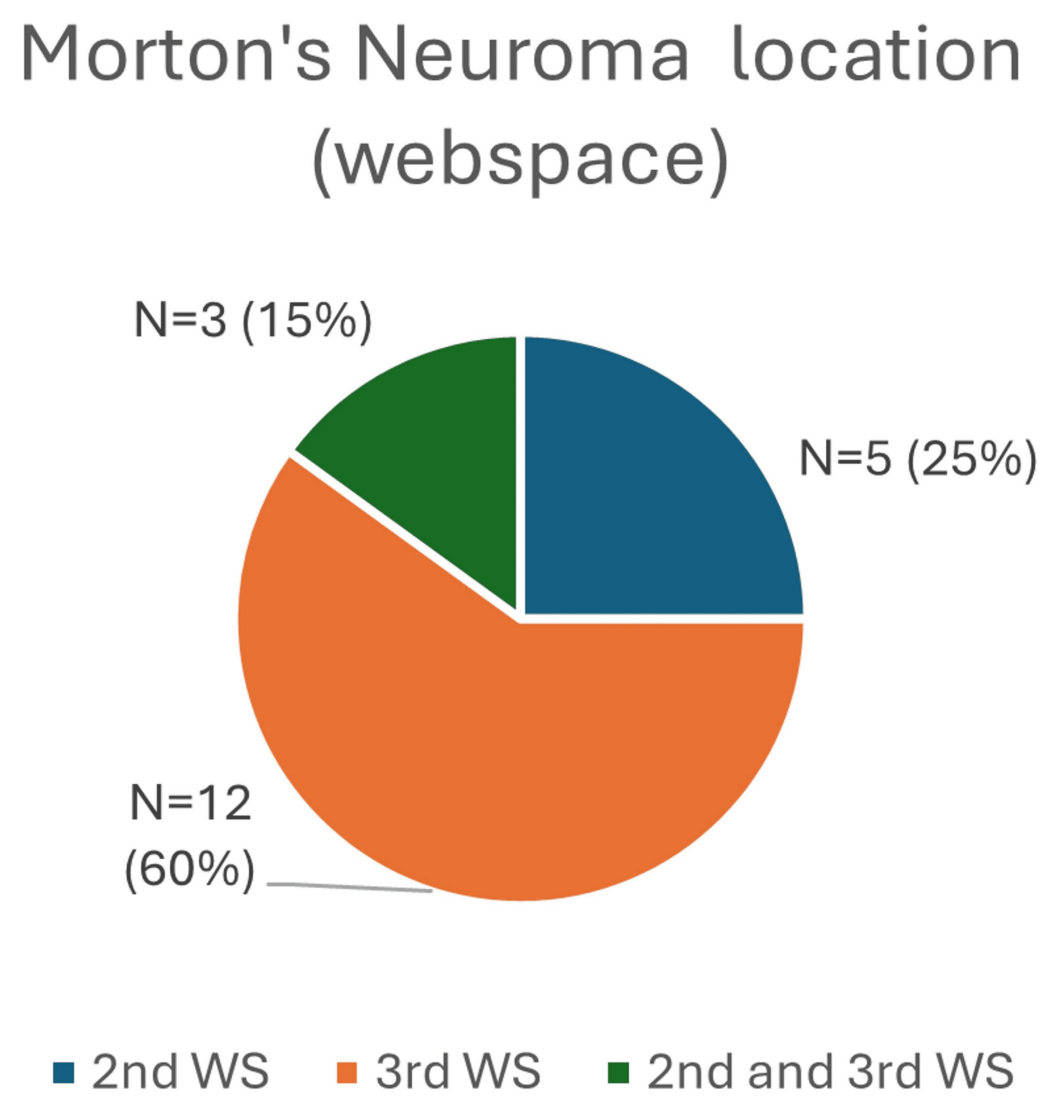
Chart showing Morton’s neuroma location WS: web space

A total of 20 patients were included, 15 (75%) were female patients and five (25%) were male patients. The age range for female patients was 28-76 years (mean: 59.1 years), and for male patients, it was 35-75 years (mean: 54.4 years). Left-sided Morton’s neuroma was present in 12 (60%) patients, whereas seven (35%) had a right-sided neuroma, and one (5%) patient had bilateral neuromas (Figure 2). The lesion locations were as follows: five (25%) patients in the second web space, 12 (60%) patients in the third web space and three (15%) patients in both the second and third web spaces on the same side (Figure 3). Six patients were current cigarette smokers or self-reported previous heavy smoking.

VAS pain scores

Before the injection, the VAS pain score ranged from 2 to 10, with a mean score of 7.7, indicating moderate to severe average pain. After the injection, the VAS pain score ranged from 0 to 9, with a mean score of 2.2, indicating significant pain relief with injection. Seventeen (85%) patients reported a reduction within a few weeks following their injection, with 50% of patients experiencing complete resolution of pain (score of 0). Three (15%) patients reported no change in pain levels. No patients reported increased VAS pain scores following corticosteroid injection.

Statistical analysis via the Wilcoxon signed-rank test to compare the scores before and after injection revealed a significant reduction in pain levels for most patients following corticosteroid injection for Morton’s neuroma (P=0.006). The median difference was 5 points, with a 95% confidence interval (CI) of 3-7.

**Figure 4:**
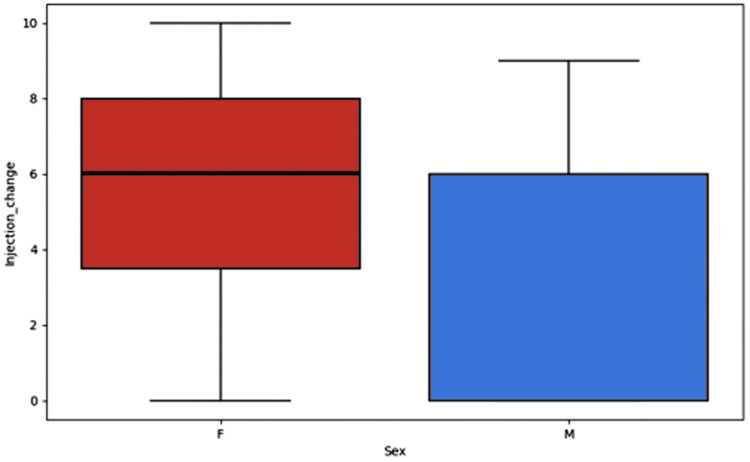
Box plot displaying the reduction in VAS pain score by sex VAS: visual analogue scale

A comparison of pre-injection and post-injection VAS pain score stratified by sex revealed a greater reduction in pain in female patients than in male patients (Figure 4). However, when this was analysed statistically with a t-test, it was not significant (P=0.33).

Size of neuromas in relation to the outcomes

The size of the neuromas on US ranged from 2.6 mm to 8.2 mm, with a mean diameter of 4.3 mm. The patient with the smallest reported neuroma (2.6 mm) had a pre-injection VAS pain score of 9, whereas the patient with the largest neuroma (8.2 mm) had a pre-injection VAS pain score of 8, suggesting that there was no correlation between neuroma size and pain levels.

We used pairwise Pearson’s correlations to test whether the size of the neuroma or the pre-injection VAS score affected the outcome of the injections. This revealed that the size of the neuroma on US and the pre-injection VAS score did not affect the outcome of the injections (P=0.767).

Duration of pain relief, number of injections and surgeries post-injection

The duration of pain relief provided by the injection varied from two weeks to three years, or to complete resolution. There was no correlation between the duration of symptom relief and the size of Morton’s neuroma, sex or smoking status.

**Figure 5:**
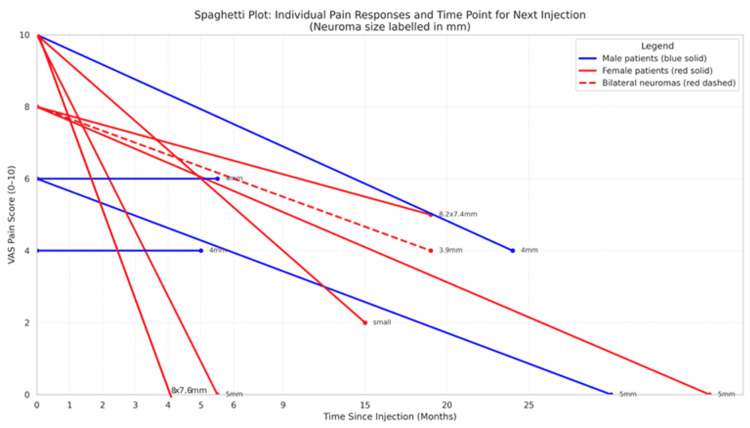
Spaghetti plot illustrating the time until repeated corticosteroid injection for individual patients

Ten (50%) patients required two or more corticosteroid injections. The duration between corticosteroid injections ranged from four months to 36 months. The time between injections with corresponding VAS scores is illustrated in Figure 5, displayed according to sex and size of the Morton’s neuroma in millimetres. Additionally, five (25%) patients eventually underwent surgical removal of their Morton’s neuroma, with another patient scheduled for surgery. Two of these six patients recorded no changes between their pre-injection and post-injection VAS pain scores. The remaining four (20%) patients who underwent surgery had severe pre-injection VAS pain scores (a score of more than 7).

## Discussion

The outcomes of this study were as follows: US-guided corticosteroid injections are 85% effective in controlling pain associated with Morton’s neuroma, with relief ranging from four months to 36 months or complete resolution. The size of the neuroma at the time of the US scan was not related to pain severity or the reduction in pain. A total of six (30%) of patients ultimately required downstream surgery, which indicated recurrence or severe pain.

Corticosteroid injections are a treatment modality that can be performed in an outpatient setting, offering greater accessibility than surgical alternatives such as neuronal excision. With the average UK theatre cost exceeding £1,200 per hour, clinicians may look towards corticosteroid injections as a safe and cost-effective option [12].

A recent systematic review by Choi et al. (2021) on corticosteroid injection and Morton’s neuroma reported findings similar to our study. Their outcomes revealed that corticosteroids provided satisfactory results in most studies, with pain reduction ranging from one week to three months. In their study, 29.85% of the subjects eventually underwent surgery [13]. This number is almost identical to that in our study, in which 25% of patients underwent surgery, and a further participant is listed for the future, which results in a total percentage of 30% of patients requiring downstream surgery.

A limitation of our data is the absence of a functional outcome measure and, therefore, the lifestyle impact of the treatment [14]. Thomson et al. (2013) demonstrated, in a patient-blinded randomised study of 131 patients, an improvement in patient assessment of Global Foot Health at three months with corticosteroid and anaesthetic injection use, compared with anaesthetic alone (P=0.002). Interestingly, there was also no influence of neuroma size [15].

A small-scale study by Sharp et al. (2009) demonstrated no correlation between the severity of symptoms and the size of the neuroma in 29 participants [16]. Moreover, Mahadevan et al. (2016) demonstrated no relationship with neuroma size in 36 patients and those who were pain-free at one year following corticosteroid injection [17].

The current literature consistently indicates that the incidence of Morton’s neuroma is greater in the female population than in the male population, and our study agrees with this finding [2,18].

In this study, there were no correlations between the size of the neuroma and the resulting pain scores following US-guided corticosteroid injections. Eight out of 14 subjects with a neuroma ≥ 4 mm required further injections. In contrast, five out of six subjects with a neuroma of ≤4 mm required only a single injection. Additionally, of the six subjects who underwent surgical excision of their neuroma, four had a neuroma size of ≥4 mm. This group also reported either an initial severe VAS pain score or no changes to their scores following corticosteroid injections. Therefore, these cohorts of patients may benefit from earlier surgical resection [19].

Interestingly, in our study, there appeared to be greater pain reduction in our female cohort than in the male cohort following corticosteroid injection. Although these findings were not statistically significant, further research is needed to understand why these results occurred.

The retrospective design of the study carries an inherent risk of selection and information bias. To reduce this, all cases were identified from a single database with consistent diagnostic and inclusion criteria. Recall bias was minimised by using a structured questionnaire and confirming information against clinical records where available.

A limitation of our study is the small sample size of patients who received US-guided corticosteroid injections for symptomatic Morton’s neuroma. In part, this was due to the overlap of the study date with the COVID-19 pandemic, during which elective clinics and procedures were either delayed or cancelled. Further studies with a larger patient cohort over a longer study period could potentially assess whether the observations and descriptive findings are statistically significant.

## Conclusions

Corticosteroid injections are safe, effective and reasonable treatment options for Morton’s neuroma, with variable durations of relief. No complications were observed in this study group following the intervention.

Further research using a larger patient cohort is needed. This is to determine whether there are any associations between the efficacy of pain reduction in female patients and that in male patients. It will also assess whether neuroma size may be related to corticosteroid treatment response and highlight those who may benefit from earlier surgery. This will improve patient experience, reduce unnecessary treatments or interventions and reduce financial costs.

## References

[1] (2024). National Institute for Health and Care Excellence: Clinical knowledge summaries: Morton’s neuroma. https://cks.nice.org.uk/topics/mortons-neuroma/background-information/definition/.

[2] Matthews BG, Hurn SE, Harding MP, Henry RA, Ware RS (2019). The effectiveness of non-surgical interventions for common plantar digital compressive neuropathy (Morton's neuroma): a systematic review and meta-analysis. J Foot Ankle Res.

[3] Jain S, Mannan K (2013). The diagnosis and management of Morton's neuroma: a literature review. Foot Ankle Spec.

[4] Adams WR 2nd (2010). Morton's neuroma. Clin Podiatr Med Surg.

[5] Valisena S, Petri GJ, Ferrero A (2018). Treatment of Morton's neuroma: a systematic review. Foot Ankle Surg.

[6] Bhatia M, Thomson L (2020). Morton's neuroma - current concepts review. J Clin Orthop Trauma.

[7] Munir U, Tafti D, Morgan S (2024). Morton neuroma. StatPearls.

[8] Markovic M, Crichton K, Read JW, Lam P, Slater HK (2008). Effectiveness of ultrasound-guided corticosteroid injection in the treatment of Morton's neuroma. Foot Ankle Int.

[9] Delgado DA, Lambert BS, Boutris N, McCulloch PC, Robbins AB, Moreno MR, Harris JD (2018). Validation of digital visual analog scale pain scoring with a traditional paper-based visual analog scale in adults. J Am Acad Orthop Surg Glob Res Rev.

[10] Jensen MP, Chen C, Brugger AM (2003). Interpretation of visual analog scale ratings and change scores: a reanalysis of two clinical trials of postoperative pain. J Pain.

[11] (2025). National Institute for Care and Health Excellence: Clinical knowledge summaries: palliative cancer care: assessment of pain. https://cks.nice.org.uk/topics/palliative-cancer-care-pain/management/assessment-of-pain/.

[12] (2025). NHS Improvement: Operating theatres: opportunities to reduce waiting lists. https://improvement.nhs.uk/resources/operating-theatres-opportunities-reduce-waiting-lists/.

[13] Choi JY, Lee HI, Hong WH, Suh JS, Hur JW (2021). Corticosteroid injection for Morton’s InterDigital neuroma: a systematic review. Clin Orthop Surg.

[14] Le D, Martin K, Clark SC, Ruso D, Hoyen A, Hunter K, Kim TW (2024). Trends in functional outcome measures in orthopedic oncology. J Orthop Res.

[15] Thomson CE, Beggs I, Martin DJ (2013). Methylprednisolone injections for the treatment of Morton neuroma: a patient-blinded randomized trial. J Bone Joint Surg Am.

[16] Sharp RJ, Wade CM, Hennessy MS, Saxby TS (2003). The role of MRI and ultrasound imaging in Morton's neuroma and the effect of size of lesion on symptoms. J Bone Joint Surg Br.

[17] Mahadevan D, Attwal M, Bhatt R, Bhatia M (2016). Corticosteroid injection for Morton's neuroma with or without ultrasound guidance: a randomised controlled trial. Bone Joint J.

[18] Park CH, Chang MC (2019). Forefoot disorders and conservative treatment. Yeungnam Univ J Med.

[19] Dr NA (2022). Morton neuroma. QJM.

